# Alternative Diets for *Chrysomya putoria*, an Old World Screwworm Fly

**DOI:** 10.1673/031.012.4301

**Published:** 2012-03-27

**Authors:** Adriana C.P. Ferraz, Daniele L. Dallavecchia, Débora Cardoso da Silva, Rafaela Pereira de Carvalho, Renato Geraldo da Silva Filho, Valéria M. Aguiar-Coelho

**Affiliations:** ^1^ Universidade Federal Rural do Rio de Janeiro — UFRRJ, Rod. BR 465, Km 7, CEP 23890-000 Seropédica, RJ, Brazil; ^2^ Universidade Federal do Estado do Rio de Janeiro — UNIRIO, Rua Frei Caneca, 94, Centro, CEP 20211 - 040 Rio de Janeiro, RJ, Brazil

**Keywords:** blowflies, fly breeding, maggots, protein, pupae

## Abstract

The purpose of this study was to evaluate the post—embryonic development of *Chrysomya putoria* (Wiedemann 1818) (Diptera: Calliphoridae) reared on a diet of gizzard or gizzard/agar homogenate, with a diet of beef used as the control. Four replicates per treatment were performed (60 mL of each diet). The gizzard (60%), distilled water, and agar homogenate were combined in a blender. Each replicate consisted of 40 newly hatched larvae of *C. putoria* (5^th^ generation). Each glass beaker containing a diet was inserted into a larger flask containing sawdust, which was covered with a nylon cloth held in place by an elastic band. The larvae were weighed and stored in test tubes sealed with a nylon cloth and an elastic band. The average temperature, measured with a thermohygrograph, was 20.6 °C, and the average relative humidity was 67.7%. The variation in the mean weight of mature larvae and in the duration of the larval, pupal, and total stages (newly hatched larvae to imagoes) were analyzed by Student's *t*—test (α = 5%), while viability was compared by ANOVA. The sex ratio was evaluated by the chi—squared test. The average duration of the period from the larval to imago stage was 8.868 days on the beef diet, 8.676 on the gizzard diet, and 9.067 on the gizzard/agar homogenate diet. Larval survival rates on these diets were 98, 92, and 73%, respectively, while pupal viabilities were 98, 91, and 71%, respectively, and larva—to—imago viabilities were 93, 83, and 64%, respectively. The duration of the pupal period differed significantly between the blowflies reared on the beef and gizzard/agar homogenate diets. The two diets proved to be good alternatives for rearing *C. putoria*.

## Introduction

*Chrysomya putoria* (Wiedemann 1818) (Diptera: Calliphoridae) is a blowfly distributed widely throughout Brazil (Guimarães et al. 1978) since its arrival in the country on ships hailing from Africa. This fly is important because it is a potential mechanical vector of type I and II poliovirus, Coxsackie viruses, *Shigella* sp., *Salmonella* sp., *Escherichia coli*, and *Giardia lamblia*, as well as other enteric pathogens, irritants and destructive agents (Greenberg 1971, 1973; Furlanetto et al. 1984). Additionally, its larvae can produce secondary myiasis (Zumpt 1965). This blowfly is found on chicken farms (Guimarães 1984), where it breeds in liquid manure with greater than 75% moisture content (Bruno et al. 1993).

The larvae of the family Calliphoridae are detritivorous, feeding on feces or open sores, or acting as parasites (Brusca and Brusca 2007). The development rate of necrophagous insects can be affected by substances introduced through their diet (Introna et al. 2001). This may affect studies of these insects, such as those involving estimates of the post—mortem interval in forensic entomology, an analysis that is based on the period of development of these insects (Estrada et al. 2009).

Efficient diets are essential for the maintenance of large dipterous fly stocks in the laboratory, and for the production of pupae to breed the parasitoid wasp *Nasonia vitripennis* (Walker 1836), a promising biological control agent that has been the focus of several studies (Marinho 2007; Barbosa et al. 2008; Mello and Aguiar-Coelho 2009).

Beef is an efficient diet for breeding larvae of necrophagous dipterans, but it releases a disagreeable odor characteristic of decomposing organic matter and increases the possibility of secondary contamination of other insects that are attracted to it (Estrada et al. 2009; Barbosa et al. 2004), in addition to being associated with high laboratory costs. Chicken gizzard also releases putrefaction odors, but its ready availability in markets and its lower cost have prompted studies to evaluate its potential as a food substrate for maintaining stocks of necrophagous dipterans such as *Chrysomya putoria* (Wiedemann 1818). On the other hand, artificial diets allow for greater control of the effects of each ingredient, reducing the release of odors, and enabling the incorporation of other substances (i.e., for toxicity tests).

The purpose of this bioassay was to monitor the performance of newly hatched larvae of *C. putoria* reared on two natural diets (beef and gizzard) and an artificial diet (gizzard/agar homogenate).

## Materials and Methods

The blowflies were reared in the Laboratory of Diptera Studies (LED) of the Department of Microbiology and Parasitology at UNIRIO - Federal University of the State of Rio de Janeiro, where the entire experimental part of this study was conducted. The *C. putoria* colony was started with imagoes collected at the Zoological Gardens of Rio de Janeiro, located in Quinta da Boa Vista Park in São Cristóvão, RJ, Brazil. Three traps were baited with sardines, as described by Ferreira (1978), and exposed for approximately 5 hours in the morning.

Imagoes and larvae of the muscoid dipteran were collected and taken to the LED, where they were examined and identified taxonomically, as described by Mello (2003). The blowflies were reared in plastic cages with a frontal opening covered with nylon fabric and were fed with water, a solution of honey and water (50%), and beef and chicken gizzard; the latter served as both sources of protein and substrates for egg laying and maturation.

Three treatments were carried out, each with four repetitions. The first served as the control (60 g of beef brisket in each repetition); the test diets consisted of chicken gizzard (60 g of gizzard per repetition) (T1) and a diet of gizzard homogenate in an agar solution with a concentration of 65% (60 mL for each repetition) (T2).

The homogenate was prepared in a blender in a concentration of three parts of gizzard to one part distilled water. The agar solution was prepared using bacteriological agar (agar—agar Micromed, www.drmicromed.com) (1.5%), following the instructions on its packaging; it was heated to 100 °C in distilled water until its complete dissolution, whereupon it was added to the homogenate. The content was mixed in test tubes with screw caps (20 × 150 mm), vortexed for approximately two min, and sterilized in an autoclave for 15 min at 121°C. After cooling, the test tubes were vortexed once more and their contents poured into 100 mL beakers.

The other treatments (beef and gizzard) involved thawing in a refrigerator for 24 hours prior to use, and then cutting the beef and gizzard into 1 cm^3^ cubes, which were then placed in the 100 mL beakers.

Each repetition involved 40 1^st^ instar larvae of *C. putoria* of the 5^th^ generation bred in the laboratory, which were handled with a paintbrush. Each 100 mL beaker of each repetition was placed in a larger beaker (400 mL) containing sterilized sawdust to allow the larvae to pupate when mature, after abandoning the diet. These larger beakers were also closed with nylon fabric secured with elastic bands.

Daily observations were made, always at the same time (12:00). The body mass of batches of five mature larvae was recorded on an analytical balance and the larvae were then stored in test tubes closed with nylon fabric secured with elastic bands to observe the emergence of the imagoes. Records were kept of the dates of pupation, emergence of imagoes, and sexual ratio, as well as morphological anomalies in the imagoes. The temperature and local relative air humidity were recorded using a thermo—hygrograph (maximum temperature: 26 °C, mean: 20.6 °C, minimum: 17.5 °C; maximum humidity: 83%, mean: 67.7%, minimum: 49%).

The raw data were analyzed using Microsoft Excel (www.microsoft.com), while the remaining analyses were performed with the STAT program. The variation between the mean weight of larvae and the duration of the larval, pupal, and total stages (newly hatched larva to imago) were analyzed using Student's *t*—test (α = 5%). The viabilities were compared by ANOVA. The sexual ratio was tested in relation to the expected frequency, using the chi—squared test (χ^2^).

## Results

No significant difference was found in the mean individual weight of the larvae in the three treatments: *p* = 0.233 (control vs. T1), *p* = 0.599 (control vs. T2), and *p* = 0.152 (T1 vs. T2) (Table 1).

With regard to the average duration of the larval stage (days), the three treatments showed no significant difference: *p* = 0.116 (control vs. T1), *p* = 0.082 (control vs. T2), *p* = 0.631 (T1 vs. T2) (Table 2). No significant difference was found between the average duration of the pupal stage (days) of the control and treatment 1 (*p* = 0.371) and between treatment 1 and treatment 2 (*p* = 0.06), but a significant difference was found between the control and treatment 2 (*p* < 0.018) (Table 2). The three treatments also showed no significant differences in the total average duration (newly hatched larva to imago): *p* = 0.174 (control vs. T1), *p* = 0.179 (control vs. T2), *p* = 0.101 (T1 vs. T2) (Table 2).

**Figure 1.**
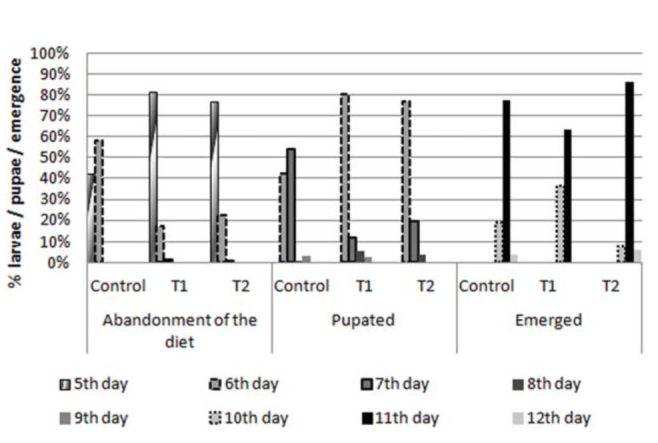
Pace, in days, at which mature larvae stopped feeding, pupated, and emerged as imagoes of *Chrysomya putoria* reared on beef (control), gizzard (T1), or gizzard/agar homogenate (T2) diets. Average temperature: 20.6 °C, average relative air humidity: 67.7%. High quality figures are available online.

**Figure 2.**
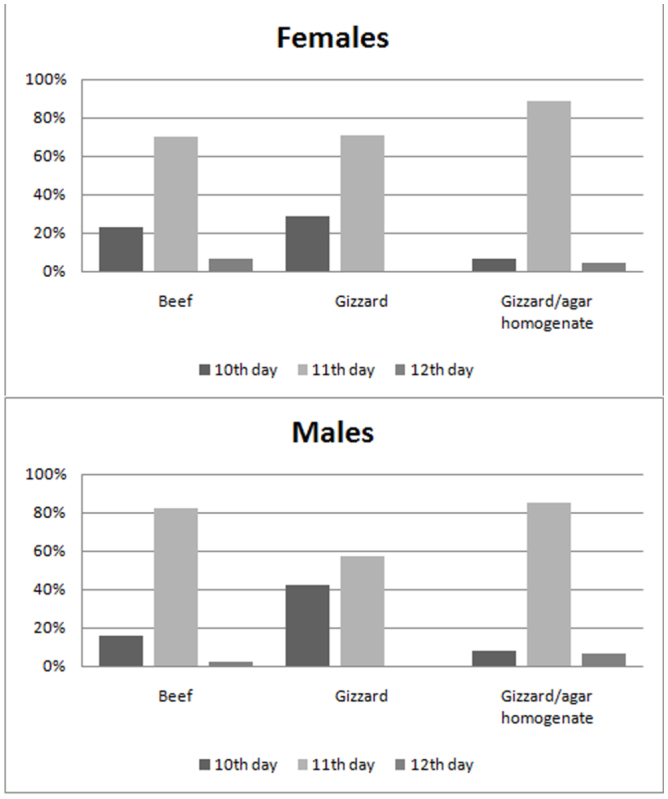
Pace of emergence, in days, of males and females of *Chrysomya putoria* reared on beef (control), gizzard (T1), or gizzard/agar homogenate (T2) diets. Average temperature: 20.6 °C, average relative air humidity: 67.7%. High quality figures are available online.

Figure 1 illustrates the pace at which mature larvae of *C. putoria* stopped feeding, pupated, and emerged as imagoes on the beef (control), gizzard (T1), and gizzard/agar homogenate (T2) diets. The T1 and T2 diets produced similar results in terms of emergence (peak on day 11), the end of feeding, and pupation. The peaks of the end of feeding and pupation of *C. putoria* reared on beef occurred one day later than on the other diets. The pace of emergence of males and females reared on the three diets (beef, gizzard, and gizzard/agar homogenate) was very similar (Figure 2).

The sexual proportions and ratios found were as follows: control (males = 58%, females = 42%, sexual ratio = 0.42), T1 (males = 55%, females = 45%, sexual ratio = 0.45), and T2 (males = 56%, females = 44%, sexual ratio = 0.44). The chi—squared tests indicated that the sexual proportions found in the three treatments did not differ from the expected ones (control: χ^2^= 3,699; T1: χ^2^ = 1,271; T2: χ^2^ = 1,412; χ^2^ indexed rate = 3.84, gl = 1, α = 5%). The three treatments produced 100% normal imagoes. 

The viability of larvae, pupae, and total for treatment 2 (gizzard/agar homogenate) differed significantly from the control: larva (control vs. T1: *p* = 0.43, control vs. T2: *p* < 0.04, T1 vs. T2: *p* = 0.17), pupa (control vs. T1: *p* = 0.373, control vs. T2: *p* < 0.05, T1 vs. T2: *p* = 0.15), and total (control vs. T1: *p* = 0.43, control vs. T2: *p* < 0.04, T1 vs. T2: *p* = 0.20). However, no significant difference was found between T1 and T2 (Table 3).

**Table 1.**
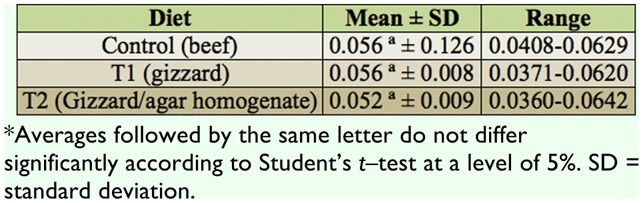
Weight of larvae (g)^*^ of *Chrysomya putoria* reared on three different diets at room temperature. Average temperature: 20.6 °C, average relative air humidity: 67.7%.

**Table 2.**
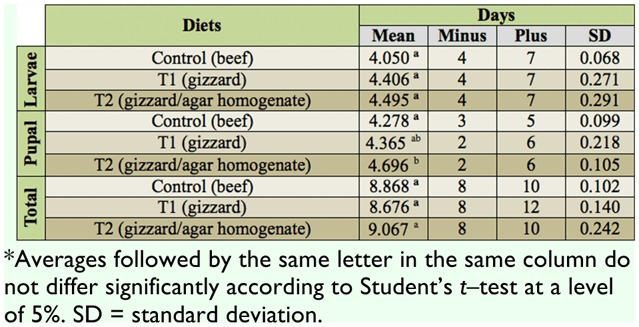
Average duration of the post—embryonic stages of development of larvae of *Chrysomya putoria* reared on three different diets (beef, gizzard, and gizzard/agar homogenate) at room temperature^*^. Average temperature: 20.6 °C, average relative air humidity: 67.7%.

**Table 3.**
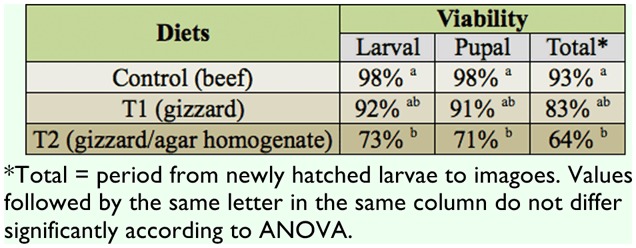
Viabilities of the larval, pupal, and total (newly hatched larva to imago) stages of *Chrysomya putoria* reared on beef, gizzard, and gizzard/agar homogenate diets^*^. Average temperature: 20.6 °C, average relative air humidity: 67.7%.

## Discussion

The quantity of food in the three repetitions was enough for 1 g of diet per larva, which, according to Aguiar-Coelho and Milward-De-Azevedo (1996), is the ideal relative density of beef diet for different calliphorids. This parameter was carefully regulated to prevent the density from interfering in the evaluation of the efficiency of these diets by causing stress through exploitative competition for resources or through chemical changes in the food substrate resulting from the larval metabolism (Khazaeli et al. 1993; Bubli et al. 1998).

D'Almeida (1994) tested several diets for *C. putoria* and found that ground beef and fish were the most efficient diets. Leal et al. (1982) reported that *C. putoria* showed greater development on rat carcasses compared with artificial oligidic diets, and noted that beef is the natural diet of these calliphorids. According to Parra (2001), for an alternative diet to be considered efficient, it must have not only the nutritional aspects that meet the insect's needs but also the physical, chemical, and microbiological characteristics that enable the feeding process. The mean weight of the larvae reared on the three tested diets did not show a significant difference in this study, possibly indicating that the alternative diets (gizzard and gizzard homogenate) were efficient because their results were similar to those of the natural diet (beef). Nevertheless, the gizzard/agar homogenate diet appeared to be more easily solubilized by the larvae than the beef and gizzard. However, this did not make it unsuitable because diets with poor solubility result in pupae of low weight due to difficulties in digesting proteins and other elements of the medium (Chaudhury et al. 2000).

The durations of the post—embryonic stages of development (larval, pupal, and total) were also equivalent in the three diets, except for the duration of the pupal stage of the control (beef) and the gizzard/agar homogenate diets. According to D'Almeida et al. (2001), diets should be evaluated according to the duration of the period from newly hatched larvae to the emergence of imagoes; this is the most efficient method because it avoids distortions between the larval and pupal periods. Thus, one can consider that the diets supplied the needs of the insects because otherwise their feeding would have been delayed or even inhibited if there were a deficiency of essential nutrients (Chippendale 1978).

In exposed rabbit carcasses, the average duration of the development of *C. putoria* was 10 days in the summer of 2005, at a mean temperature of 20 °C, in Pelotas, Rio Grande do Sul, Brazil (Kirst 2006). This was longer than the duration recorded in the present study, which varied on average from eight to nine days. However because the climate conditions were not controlled in either of these studies, it is possible that oscillations occurred during the periods of study. According to Higley and Haskell (2003), development time is dictated by temperature oscillations.

The sexual rates of the individuals reared on the three diets tested in this study indicated population stability, according to the chi—squared analysis and to the tenets of Fisher (1930), which state that a population will only show stability when the sexual ratio is 1:1; a sexual ratio with a deviation is not evolutionally stable because gradual increases in the proportion of the sex observed in lesser number will occur in future generations.

Because 100% of the dipterans were normal, we can consider that the density of dipterans in the diet was adequate for their development; this factor affects the phenotypic determination of bionomic traits and alters components that determine the adaptive value of organisms, as reported by Ribeiro et al. (1993), Reis et al. (1994), Roper et al. (1996), and Ribeiro (1998) in studies on Diptera. The absence of variations in symmetry indicates that no sufficiently intense disturbances occurred that could not be buffered or neutralized through metabolic adjustments to ensure homeostatic stability in the patterns of development (Lomonaco and Germanos 2001).

As for larval viability, it is known that some larvae eat more than the minimum required for survival, while others eat the minimum necessary, and still others are not able to ingest the minimum needed to pupate and thus die (De Jong 1976). In our study, the control and gizzard diets achieved high rates of larval, pupal, and total viabilities, while the viabilities of larvae on the gizzard/agar homogenate diet were significantly lower than those of the control, despite reaching weights equivalent to the larvae reared on the other diets. Smaller larvae are at a disadvantage in competition with larger ones, taking longer to pupate and covering longer distances to do so or to look for more food (Gomes et al. 2002). The larval viability rate in the gizzard homogenate diet was 73% (60 g of diet for 40 larvae = 1.5 g of diet per larva), presenting a similar rate to that reported by Godoy et al. (1996), who used an artificial diet, and the rate reported by Leal et al. (1982), of 73.25% at a density of 50 g of diet for 100 larvae (0.5 g of diet per larva) and at the 20 other densities tested with smaller quantities of diets fed to larvae. The diet used by Leal et al. (1982) has been used by several other authors (Von Zuben et al. 2000; Estrada et al. 2009), and the achievement of a similar viability indicates a good result.

The use of larval therapy requires knowledge of the behavior of larvae in the presence of medicines that patients with a potential for this type of treatment may be using, such as antibiotics to prevent or fight infections. The artificial diet tested here would allow for the dissolution of such medicines in order to observe potential reactions of dipterans to their presence in the food substrate. Moreover, the fact that the diet contains few ingredients is an advantage when the aim is to test the influence of chemical products on the development of insects because the insect will be less affected by the medium when they are added to this diet. Similarly, chemical substances such as medicines could also cause changes in the development of dipterans and thus alter estimates of the post—mortem interval in forensic studies. Torres (2005) notes that experiments have been performed which involved the addition of antimicrobials to artificial diets for insect larvae to prevent their contamination by microorganisms and test the activity of these drugs in larval development and maturation.

The agar base used in our artificial diet has already been employed to create diets for other insects, most of them of agricultural interest (Panizzi and Parra 1991), and was chosen as a component due to its firm gel density at temperatures close to 37 °C (Insumos 2008) in an attempt to mimic the consistency of beef. Moreover, agar is nontoxic to the majority of microorganisms and humans, is non—absorbable, non—fermentable, and its composition consists mostly of fibers and mineral salts (P, Fe, K, Cl, I), cellulose, anhydrogalactose, and a small quantity of proteins (Insumos 2008).

The authors that tested diets for calliphorids include Paes and Milward-De-Azevedo (1998), Nespoli et al. (1998), and Paes et al. (2000), who examined the effect of natural diets in different stages of decomposition on the post—embryonic period of dipterans, as well as Cunha-e-Silva and Milward-De-Azevedo (1994), Leal et al. (1982, 1991), D'Almeida et al. (2000), and Mendonça et al. (2009), who tested artificial diets. The latter diets may be advantageous due to the possibility of higher nutritional and biological uniformity of the colonies over time (Parra 2001). The gizzard/agar homogenate diet tested here proved to be efficient for this purpose. Similarly, alternative diets such as chicken gizzard can also be used due to their lower cost in comparison to beef, favoring the development of projects and research that require the maintenance of large stocks of dipterans for long periods.
